# Altitudinal variation in soil organic carbon stock in coniferous subtropical and broadleaf temperate forests in Garhwal Himalaya

**DOI:** 10.1186/1750-0680-4-6

**Published:** 2009-08-25

**Authors:** Mehraj A Sheikh, Munesh Kumar, Rainer W Bussmann

**Affiliations:** 1 Department of Forestry, HNB Garhwal University, Srinagar Garhwal, Uttarakhand, India; 2 William L. Brown Center, Missouri Botanical Garden, St. Louis, MO 63110, USA

## Abstract

**Background:**

The Himalayan zones, with dense forest vegetation, cover a fifth part of India and store a third part of the country reserves of soil organic carbon (SOC). However, the details of altitudinal distribution of these carbon stocks, which are vulnerable to forest management and climate change impacts, are not well known.

**Results:**

This article reports the results of measuring the stocks of SOC along altitudinal gradients. The study was carried out in the coniferous subtropical and broadleaf temperate forests of Garhwal Himalaya. The stocks of SOC were found to be decreasing with altitude: from 185.6 to 160.8 t C ha^-1 ^and from 141.6 to 124.8 t C ha^-1 ^in temperature (*Quercus leucotrichophora*) and subtropical (*Pinus roxburghii*) forests, respectively.

**Conclusion:**

The results of this study lead to conclusion that the ability of soil to stabilize soil organic matter depends negatively on altitude and call for comprehensive theoretical explanation

## Background

Soils are the largest carbon reservoirs of the terrestrial carbon cycle. About three times more carbon is contained in soils than in the world's vegetation and soils hold double the amount of carbon that is present in the atmosphere. Worldwide the first 30 cm of soil holds 1500 Pg carbon [1]; for India the figure is 9 Pg [2]. Soils play a key role in the global carbon budget and greenhouse effect [3]. Soils contain 3.5% of the earth's carbon reserves, compared with 1.7% in the atmosphere, 8.9% in fossil fuels, 1.0% in biota and 84.9% in the oceans [4]. The amount of CO_2 _in the atmosphere steadily increases as a consequence of anthropogenic emissions, but there is a large interannual variability caused by terrestrial biosphere [5].

The first estimate of the organic carbon stock in Indian soils was 24.3 pg (1 Pg = 10^15 ^g) based on 48 soil samples [6]. Forest soils are one of the major carbon sinks on earth, because of their higher organic matter content [7]. Soils can act as sinks or as a source for carbon in the atmosphere depending on the changes happening to soil organic matter. Equilibrium between the rate of decomposition and rate of supply of organic matter is disturbed when forests are cleared and land use is changed [8,9]. Soil organic matter can also increase or decrease depending on numerous factors, including climate, vegetation type, nutrient availability, disturbance, and land use and management practice [10,11]. Physical soil properties, such as soil structure, particle size, and composition, have profound impact on soil carbon (C). Soil particle size has an influence on the rate of decomposition of soil organic carbon [12]. The release of nutrients from litter decomposition is a fundamental process in the internal biogeochemical cycle of an ecosystem, and decomposers recycle a large amount of carbon that was bounded in the plant or tree to the atmosphere [13].

About 40% of the total SOC stock of the global soils resides in forest ecosystem [14]. The Himalayan zones, with dense forest vegetation, cover nearly 19% of India and contain 33% of SOC reserves of the country [15]. These forests are recognized for their unique conservation value and richness of economically important biodiversity. Managing these forests may be useful technique to increase soil carbon status because the presence of trees affects carbon dynamics directly or indirectly. Trees improve soil productivity through ecological and physicochemical changes that depend upon the quantity and quality of litter reaching soil surface and rate of litter decomposition and nutrient release [16].

The current global stock of soil organic carbon is estimated to be 1,500–1,550 Pg [1,17,18]. This constituent of the terrestrial carbon stock is twice that in the earth's atmosphere (720 Pg), and more than triple the stock of organic carbon in terrestrial vegetation (560 Pg) [19,20]. To sustain the quality and productivity of soils, knowledge of SOC in terms of its amount and quality is essential. The first comprehensive study of organic carbon (OC) status in Indian soils was conducted [21] by collected 500 soil samples from different cultivated fields and forests with variable rainfall and temperature patterns. However, the study did not make any estimate of the total carbon reserves in the soils. The first attempt in estimating OC stock [22] was also made based on a hypothesis of enhancement of OC level on certain unproductive soils. In last decade, the greenhouse effect has been of great concern, and has led to several studies on the quality, kind, distribution and behaviour of SOC [23,1,24]. Global warming and its effect on soils in terms of SOC management have led to several quantitative estimates for global C content in the soils [23,1-26]. Although, so far the soil organic carbon stock studies in Indian Himalayan forests in relation to altitudinal gradient are not available. Therefore, the aim of the present study is made to estimate SOC stocks of two dominant forests of subtropical (*Pinus roxburghii*) and temperate (*Quercus leucotrichophora*) along altitudinal gradient in Garhwal Himalaya.

## Results

Depth wise SOC results are mentioned in Tables 1 and 2. A decreasing trend in soil organic carbon (SOC) was observed with increased soil depths in all the sites except site-II of the *Pinus roxburghii *forest, where organic carbon was highest in the top layer (0–20 cm) and lowest in middle depth (20–40 cm). The carbon level increased again below the middle depth. In site-I of the *Quercus leucotrichophora *forest, the level of soil organic carbon ranged from 24.3 ± 1.9 g kg^-1 ^to 21.9 ± 3.1 g kg^-1 ^and was higher in the upper layer, dropping with an increase in depth. The trend was same for site-II and site-III where the SOC values also decreased with increasing depths, and ranged from 23.4 ± 0.8 g kg^-1 ^to 21.9 ± 1.2 g kg^-1 ^and 22.5 ± 2.6 g kg^-1 ^to 16.5 ± 2.1 g kg^-1^, respectively. The range of soil organic carbon in *Pinus roxburghii *forest was 18.0 ± 6.5 g kg^-1 ^to 12.1 ± 0.9 g kg^-1^, 19.6 ± 0.9 g kg^-1 ^to 11.2 ± 0.3 g kg^-1 ^and 19.6 ± 0.5 g kg^-1 ^to 15.0 ± 0.2 g kg^-1 ^for site-I, site-II, and site-III, respectively, again the levels were higher in the top layer and decreased with depth.

**Table 1:** SOC (± SD) values at different depths of *Quercus leucotrichophora *forest soils

**Site**	**Soil depth (cm)**	**SOC g kg^-1^**
Site-I	0–20	24.3 ± 1.9
	20–40	23.4 ± 3.4
	40–60	21.9 ± 3.1
Site-II	0–20	23.4 ± 0.8
	20–40	22.5 ± 3.3
	40–60	21.9 ± 1.2
Site-III	0–20	22.5 ± 2.6
	20–40	21.5 ± 6.8
	40–60	16.5 ± 2.1

**Table 2:** SOC (± SD) values at different depths in *Pinus roxburghii *forest soils

**Site**	**Soil depth (cm)**	**SOC g kg^-1^**
Site-I	0–20	18.0 ± 6.5
	20–40	16.8 ± 4.1
	40–60	12.1 ± 0.9
Site-II	0–20	19.6 ± 0.9
	20–40	11.2 ± 0.3
	40–60	16.8 ± 5.3
Site-III	0–20	18.0 ± 6.5
	20–40	18.7 ± 8.4
	40–60	15.0 ± 0.2

The maximum carbon stock was present in *Quercus leucotrichophora *forest soils. The higher percent of soil organic carbon in *Quercus leucotrichophora *forest may be due to dense canopy and higher input of litter which results in maximum storage of carbon stock. In *Quercus leucotrichophora *forest sites dense vegetation led to higher accumulation of soil organic carbon as compared to coniferous sites. In *Pinus roxburghii *forest, the lower amount of organic carbon might be due to wider spacing between trees, resulting in lower litter input and less accumulation, in turn yielding less storage of carbon stock in these forest soils.

In *Quercus leucotrichophora *forest soils (Table 3), the maximum carbon stock was present in site-I (185.6 t C ha^-1^) and minimum in site-III (160.8 t C ha^-1^). The trend was the same for the *Pinus roxburghii *forest soils (Table 4), where the highest carbon stock was present in site-I (141.6 t C ha^-1^) followed by site-II (126.4 t C ha^-1^) and site-III (124.8 t C ha^-1^). While comparing the soil organic carbon stock values of different sites with each other in both forests, the carbon stock tended to decrease with increasing altitudes. In the present study, a characteristic decline in vegetation was observed across altitudinal strata and among sites. Altitude had a significant effect on species richness, which declines with even a 100 m increase in altitude. The characteristic decline in vegetation with increasing altitude results in less accumulation of litter and low input of organic carbon in soils.

**Table 3:** Soil Organic Carbon stock (up to 60 cm depth) in *Quercus leucotrichophora *forest

**Site**	**Altitudinal range**	**SOC g kg^-1^**	**Carbon stock (t C ha^-1^)**
Site-I	1,600–1,800 m	23.2 ± 1.2	185.6
Site-II	1,800–2,000 m	22.6 ± 0.7	180.8
Site-III	2,000–2,200 m	20.1 ± 3.2	160.8

**Table 4:** Soil Organic Carbon stock (up to 60 cm depth) in *Pinus roxburghii *Forest

**Site**	**Altitudinal range**	**SOC (%)**	**Carbon stock (t C ha^-1^)**
Site-I	600–800 m	17.7 ± 0.24	141.6
Site-II	800–1,000 m	15.8 ± 0.42	126.4
Site-III	1,000–1,200 m	15.6 ± 0.31	124.8

## Discussion

The soil organic carbon (SOC) decreased with increasing soil depths in all the sites except site-II of the *Pinus roxburghii *forest, where organic carbon was highest in the top layer (0–20 cm) and lowest in middle depth (20–40 cm). In the *Quercus leucotrichophora *forest, for all sites (site-I, site-II and site-III) the level of soil organic carbon was higher in the upper layer, dropping with an increase in depth. The similar trend (higher in top layer and decreased with increasing depths) of soil organic carbon is also reported in the *Pinus roxburghii*. The higher organic carbon content in the top layer may be due to rapid decomposition of forest litter in a favorable environment. SOC represents [27] a significant pool of carbon within the biosphere. Climate shifts in temperature and precipitation have a major influence on the decompositions and amount of SOC stored within on ecosystem and that released into the atmosphere. The rate of cycling of carbon at different depths and in different pools across different vegetal cover is still not clear. There is not, as yet, enough information to predict how the size and residence time of different fractions of soil organic carbon varies [28]. The higher concentration of soil organic carbon in top layer has also been reported by various authors [28,29]. The steep fall in the SOC content as depth increases is an indication of higher biological activity associated with top layers. Our results are in accordance with earlier studies [28,30].

The maximum carbon stock was present in *Quercus leucotrichophora *forest soils. The higher percent of soil organic carbon in *Quercus leucotrichophora *forest may be due to dense canopy and higher input of litter which results in maximum storage of carbon stock. In *Quercus leucotrichophora *forest sites dense vegetation led to higher accumulation of soil organic carbon as compared to coniferous sites. The higher accumulation of soil organic carbon found in maquis vegetation, as opposed to coniferous forest, has been reported by [31]. In *Pinus roxburghii *forest, the lower amount of organic carbon might be due to wider spacing between trees, resulting in lower litter input and less accumulation, in turn yielding less storage of carbon stock in these forest soils. The study of [32] indicated a positive influence of residue application on soil carbon content. The added litter [33] and the proliferated root system [34] of the growing plants probably influenced the carbon storage in the soil, suggesting a positive correlation of SOC with the quantity of litter fall [35]. The study [36] suggested that coarse and fine woody debris are substantial forest ecosystem carbon stock. The production and decay rate of forest woody detritus depends partially on climatic conditions. The results of this study indicated that highest carbon stock founding region with cool summer, while lower carbon in arid desert/steppes or temperate humid regions.

In *Quercus leucotrichophora *forest soils (Table 3), the maximum and minimum values of carbon stock was 185.6 t C ha^-1 ^(site-I) and 160.8 t C ha^-1 ^(site-III) respectively. The trend was similar for the *Pinus roxburghii *forest soils (Table 4), where the highest and lowest values of carbon stock was 141.6 t C ha^-1 ^(site-I) and 124.8 t C ha^-1 ^(site-III). A study of [37] recorded the following levels of organic carbon stored in some Indian soils: 41.2 t C ha^-1^, 120.4 t C ha^-1^, 13.2 t C ha^-1^, and 18 t C ha^-1 ^in the Red soil, Laterite soil, Saline soil and Black soil respectively; all these measurements were lower than in the present study. Another study showed [3] the national average content of soil organic carbon was 182.94 t C ha^-1^. The total amount of soil organic carbon stored in *Quercus leucotrichophora *forest soils is almost similar to the national average and expresses the excellent ability of these forests to stock and sequester organic carbon. However, the total amount of organic carbon stored in *Pinus roxburghii *forest soils was lower than the national average.

A study carried of grassland in two different sites i.e., Mehrstedt and Kaltenborn, where SOC stocks at the clay rich Mehrstedt site were almost twice as high as at the sandy Kaltenborn site [38]. The clay soil texture was contained on average 123 t C ha^-1 ^for 0–60 cm depth. A compilation of 121 soil profiles of temperate grasslands, mainly from North America from several databases, resulted in a mean carbon stock of 91 t C ha^-1 ^for 0–60 cm depth [39]. However, the range of carbon stocks in temperate grasslands may be between 30 and 80 t C ha^-1 ^[40]. Soil organic matter (SOM) is a major component of global carbon cycle [41], increases with precipitation and decreases with temperature [42-44]. SOM content were also reported in the top 0–50 cm soil layer is positively correlated with the precipitation/temperature ratio in the Pampa and Chaco soils in Argentina [45].

While comparing the soil organic carbon stock values of different sites with each other in both forests, the carbon stock tended to decrease with increasing altitudes. A soil carbon study in Kathmandu valley of Nepal in *Pinus roxburghii *forest along altitudinal gradient at an elevation ranging between 1, 200 to 2,200 reported that the higher altitude soil was found to be much more depleted of C than the lower altitude soil [46]. The decreasing trend of C might be attributed to the lower mineralization rate and net nitrification rate at the higher altitude. A study carried out [47] in Himalayan forests indicates a characteristic decline in total tree density and basal area was apparent with increasing altitude. In the present study, a characteristic decline in vegetation was observed across altitudinal strata and among sites. The decrease in species richness in high elevation strata could be due to eco-physiological constraints, low temperature and productivity [48]. Altitude had a significant effect on species richness, which declines with even a 100 m increase in altitude. Species composition too is significantly affected by altitude [49]. Altitude is often employed to study the effects of climatic variables on SOM dynamics [50,44]. Temperature decreased and precipitation increased with increasing altitude. The changes in climate along altitudinal gradients influence the composition and productivity of vegetation and, consequently, affect the quantity and turnover of SOM [50,51]. Altitude also influences SOM by controlling soil water balance, soil erosion and geologic deposition processes [52]. The advantages of altitudinal gradients in forest soil for testing the effects of environmental variables on SOM dynamics is emphasized [50]. The relationship between SOM and altitude has also been investigated and positive correlations were reported [53,54]. A study of wetland, the balance between carbon input (organic matter production) and output (decomposition, methanogenisis, etc.) and the resulting storage of carbon depend on topography and the geological position of wetland; the hydrological regime; the type of plant present; the temperature and moisture of the soil; pH and the morphology [55]. There is a strong relation between climate and soil carbon pools where organic carbon content decreases with increasing temperatures, because decomposition rates doubles with every 10°C increase in temperature [41].

The characteristic decline in vegetation with increasing altitude results in less accumulation of litter and low input of organic carbon in soils. Similar findings were also reported [13]; the number of trees per hectare decreases with increasing elevation, the comments related to kg ha^-1 ^unquestionable give consequences implying that all weight parameters decreases at the altitude increases. A study carried out in the Western Ghats of southern India also shows the decline of soil organic carbon from 110.2 t C ha^-1 ^at an elevation > 1400 m to 82.6 t C ha^-1 ^at an elevation > 1800 m [56]. The increasing tendency of carbon density with decreasing altitude may be better stabilization of SOC at lower altitudes. It is a proven fact that forest ecosystems are the best way to sequester carbon; however, considering the huge human population in developing country like India, much of the land cannot be spared for increase in forest cover. In such circumstance the management of vast areas of Himalayan forests at lower elevations can be regarded as major sinks of mitigating atmospheric carbon dioxide. Forests at higher altitudes can be seen as potential carbon sinks.

## Methods

The study area is situated in Tehri Garhwal, one of the western-most districts of the Uttarakhand State, and located on the outer ranges of the mid-Himalayas, which comprise low line peaks rising directly from the plains of the northern India. The study site lies between 30° 18' 15.5" and 30° 20' 40" N latitude and 78° 40' 36.1" to 78° 37' 40.4" E longitude. Three sites were selected within *Pinus roxburghii *forest at an altitude of 700 m (site-I), 900 m (site-II), 1100 m asl (site-III) and three sites in *Quercus leucotrichophora *forest at altitudes of 1700 m (site-I), 1900 m (site-II) and 2100 m (site-III).

The quality of organic carbon data of the soils depends on sampling methods, the kind of vegetation, and the method of soil analysis performed in the laboratory. The sampling was done by nested plot design method. In each site, a plot of 100 × 20 m size was laid, and six sampling points were selected in each plot by the standard method [57]. Three samples were collected at each sampling point at three depths (0–20, 20–40, 40–60 cm). A total of 108 soil samples (18 from each site) were collected by digging soil pits (6 × 3 × 6 cm). The soil samples were air dried and sieved (< 2 mm) before analysis. Soil organic carbon for various depths was determined by partial oxidation method [58]. Soil samples from each depth were analysed, however to express the total SOC stock data in 0–20, 20–40, 40–60 cm, the weighted mean average were considered. The total SOC stock was estimated by multiplying the values of SOC g kg^-1 ^by a factor of 8 million, based in the assumption that a layer of soil 60 cm deep covering an area of 1 ha weighs 8 million kg [7].

## Conclusion

A comparison of the soil organic carbon stock values of different sites in both forests show that the carbon stock tonnes per hectare decrease with increasing altitudes. The tendency of carbon density to increase as altitude decreases may be due to better stabilization of SOC at lower altitudes. Considering the huge human population in developing country like India, much of the land cannot be spared for increase in forest cover. In such circumstance the management of vast areas of Himalayan forests at lower elevations can be regarded as major sinks of mitigating atmospheric carbon dioxide.

## Competing interests

The authors declare that they have no competing interests.

## Authors' contributions

MAS and MK share the contributions to fieldwork, data analysis, and compilation of this manuscript. RWB shares his valuable contribution from initial manuscript drafting to final submission. All authors read and approved the final manuscript.
